# CoronaVac vaccine is effective in preventing symptomatic and severe COVID-19 in pregnant women in Brazil: a test-negative case-control study

**DOI:** 10.1186/s12916-022-02353-w

**Published:** 2022-04-05

**Authors:** Enny S. Paixao, Kerry L. M. Wong, Flavia Jôse Oliveira Alves, Vinicius de Araújo Oliveira, Thiago Cerqueira-Silva, Juracy Bertoldo Júnior, Tales Mota Machado, Elzo Pereira Pinto Junior, Viviane S. Boaventura, Gerson O. Penna, Guilherme Loureiro Werneck, Laura C. Rodrigues, Neil Pearce, Mauricio L. Barreto, Manoel Barral-Netto

**Affiliations:** 1 London School of Hygiene and Tropical Medicine, London, WC1E 7HT UK; 2 Center of Data and Knowledge Integration for Health (CIDACS), Gonçalo Moniz Institute, Oswaldo Cruz Foundation, Salvador, Bahia Brazil; 3 LIB and LEITV Laboratories, Instituto Gonçalo Moniz, Fiocruz, Salvador, Bahia Brazil; 4 Universidade Federal da Bahia, Salvador, Bahia Brazil; 5 Universidade Federal de Ouro Preto, Ouro Preto, Brazil; 6 Tropical Medicine Centre, Fiocruz School of Government Brasília, University of Brasília, Brasília, Brazil; 7 Instituto de Medicina Social, Universidade do Estado do Rio de Janeiro, Rio de Janeiro, Brazil; 8 Instituto de Estudos em Saúde Coletiva, Universidade Federal do Rio de Janeiro, Rio de Janeiro, Brazil

**Keywords:** Vaccine effectiveness, Inactivated vaccine, Pregnant women, COVID-19, SARS-CoV-2

## Abstract

**Background:**

More doses of CoronaVac have been administered worldwide than any other COVID-19 vaccine. However, the effectiveness of COVID-19 inactivated vaccines in pregnant women is still unknown. We estimated the vaccine effectiveness (VE) of CoronaVac against symptomatic and severe COVID-19 in pregnant women in Brazil.

**Methods:**

We conducted a test-negative design study in all pregnant women aged 18–49 years with COVID-19-related symptoms in Brazil from March 15, 2021, to October 03, 2021, linking records of negative and positive SARS-CoV-2 reverse transcription polymerase chain reaction (RT-PCR) tests to national vaccination records. We also linked records of test-positive cases with notifications of severe, hospitalised or fatal COVID-19. Using logistic regression, we estimated the adjusted odds ratio and VE against symptomatic COVID-19 and against severe COVID-19 by comparing vaccine status in test-negative subjects to test-positive symptomatic cases and severe cases.

**Results:**

Of the 19,838 tested pregnant women, 7424 (37.4%) tested positive for COVID-19 and 588 (7.9%) had severe disease. Only 83% of pregnant women who received the first dose of CoronaVac completed the vaccination scheme. A single dose of the CoronaVac vaccine was not effective at preventing symptomatic COVID-19. The effectiveness of two doses of CoronaVac was 41% (95% CI 27.1–52.2) against symptomatic COVID-19 and 85% (95% CI 59.5–94.8) against severe COVID-19.

**Conclusions:**

A complete regimen of CoronaVac in pregnant women was effective in preventing symptomatic COVID-19 and highly effective against severe illness in a setting that combined high disease burden and marked COVID-19-related maternal deaths.

**Supplementary Information:**

The online version contains supplementary material available at 10.1186/s12916-022-02353-w.

## Background

During pregnancy, cardiopulmonary and immune changes induce shifts in immune responses, increasing pregnant women’s susceptibility to some infectious-related adverse outcomes [1]. Although pregnant women have a higher risk of COVID-19 complications, need intensive care and mechanical ventilation and have a higher fatality [2], they were excluded from most COVID-19 vaccine trials [3]. There is considerable interest in establishing the safety and efficacy/effectiveness of COVID-19 vaccines in this population [4]. Several observational studies of vaccine effectiveness (VE) were recently conducted [5–8], but those studying pregnant women were restricted to mRNA vaccines [9–13].

More doses of CoronaVac, an inactivated virus vaccine, have been administered than any other COVID-19 vaccine, mainly in low- and middle-income countries due to logistic constraints of cold chain and lower cost compared to mRNA vaccines. Many low- and middle-income countries are conducting vaccination campaigns using CoronaVac [5], and some countries, like Brazil, offer CoronaVac to pregnant women. On January 17, 2021, the Brazilian Ministry of Health initiated COVID-19 vaccination with two CoronaVac doses and 2 to 4 weeks between doses (Additional file 1: Table S1). The policy followed internationally agreed priorities [14]. On March 15, 2021, pregnant women with co-morbidities and in occupations considered, on balance, to be at high risk became eligible to receive COVID-19 vaccine [15]. On April 26, this recommendation was expanded to include all pregnant women [16]. Although the exact figures for pregnant women are unclear, we anticipated that enough pregnant women would have been vaccinated to make it possible to evaluate vaccine effectiveness in pregnant women: Brazil combines a sufficient vaccine coverage (more than 50% of the population with two doses) [17], more than 21 million cases and 600,000 deaths (October 2021) [18], and a considerable number of maternal deaths [19, 20].

In this observational study of routine data in Brazil, we estimated the vaccine effectiveness (VE) of the CoronaVac vaccine against symptomatic and against severe COVID-19 in pregnant women using a test-negative design (TND).

## Methods

### Objectives and study design

The primary objective of this study was to estimate the VE of the CoronaVac vaccine against COVID-19 in pregnant women. We did that by conducting a test-negative design (TND) in all pregnant women in Brazil who had an RT-PCR test for COVID-19. We estimated the effectiveness of the vaccine against symptomatic COVID by comparing women’s vaccine status in symptomatic women with a positive test to vaccine status in those with a negative test and the effectiveness against severe COVID-19 (by vaccine status in those with severe, hospitalised or fatal COVID-19 with the vaccine status of those with a negative test).

### Data sources

All data used was abstracted from 3 routinely collected sources: the national surveillance system for RT-PCR test for COVID-19 (e-SUS Notifica), the information system for severe acute respiratory illness (SIVEP-Gripe) and the national immunisation system (SI-PNI).

#### e-SUS Notifica

This database contains information on all suspected cases of COVID-19 recorded in the country. It includes all positive and negative RT-PCR test results and information on residence, demographic and clinical data of individuals, such as the presence of co-morbidities and pregnancy status (so we can identify women registered during pregnancy) and presence of symptoms, with acute respiratory diseases defined as the presence of at least two of the following signs and symptoms: fever (even if referred), chills, sore throat, headache, cough, runny nose and loss or change to a sense of smell or taste [21]. Asymptomatic individuals with an RT-PCR test were not included in this study, independent of the test result.

#### SIVEP-Gripe

SIVEP-Gripe is the national register for severe acute respiratory syndrome (SARS) in Brazil, created after the influenza pandemic of 2009. In 2020, it was expanded to include COVID-19. All COVID-19 hospitalisations and deaths are meant to be registered in this system [22]. In SIVEP-Gripe, severe acute respiratory illness is defined as an individual with acute respiratory disease who presents dyspnea/respiratory discomfort, persistent pressure or pain in the chest and oxygen saturation less than 95% without oxygen or cyanosis of the lips or face [22]. Individuals who died with severe acute respiratory illness independent of hospitalisation are also registered. By linking these data with e-SUS Notifica, we identified which pregnant women in e-SUSNotify with a positive RT-PCR test progressed to severe disease.

#### SI-PNI

SI-PNI contains data on all vaccines administered in Brazil. COVID-19 vaccines are administered by health services and recorded in point-of-care applications [23]. From SI-PNI, we extracted information on which COVID-19 vaccine was received with first and second doses dates. By linking these data with the data on pregnant women in the other files, we were able to determine (i) which pregnant women who tested negative for COVID-19 had been vaccinated, (ii) which pregnant women with confirmed symptomatic COVID-19 infections had been vaccinated and (iii) which pregnant women with severe COVID-19-associated severe case had been vaccinated. We assumed that pregnant women whose records did not link to a SI-PNI vaccination record were not vaccinated.

All data were extracted on October 05, 2021, and made available by the Brazilian Ministry of Health. The information technology bureau of the Brazilian Ministry of Health provided pseudo-anonymised data with a common unique identifier that were used to link individual-level records from the three databases (more details about linkage procedures are available at https://vigivac.fiocruz.br/).

### Study population

All pregnant women with symptoms suggesting COVID-19, aged between 18 and 49 years in Brazil with a record of an RT-PCR test between March 15, 2021, and October 03, 2021, registered in e-SUS Notifica. Variants of concern have played an important role in the SARS-CoV-2 pandemic in Brazil. From March to July 2021, the gamma variant accounted for most of the SARS-CoV-2 isolates genotyped in Brazil; from August to October, the delta variant was predominant [24].

Testing for COVID-19 in Brazil is accessible to anyone through the universal public health system (SUS). Subjects who received any other COVID-19 vaccine were excluded: ChAdOx1 nCoV-19 or Ad26.COV2.S (Janssen/Johnson & Johnson) because these are not indicated for pregnant women in Brazil, and BNT162b2 (Pfizer) because numbers of women with complete regimen were too small to allow evaluation given they were only included in the Brazilian program more recently, and the long interval between doses. As a result, the study is restricted to evaluating CoronaVac vaccine effectiveness. The population consisted of symptomatic pregnant women who were tested with RT-PCR for COVID-19 classified into 3 groups: RT-PCR test negative, RT-PCR test positive with COVID-19 symptoms and RT-PCR test positive with severe COVID-19. The study population in the TND included all symptomatic women with an RT-PCR irrespective of the test result.

### Definition of outcome, cases and controls

The primary outcome was a positive RT-PCR test in a symptomatic subject. Cases were defined as all symptomatic women in the study population with an RT-PCR test result from a respiratory sample collected within 10 days after the onset of symptoms and who did not have a positive RT-PCR test result in the preceding 90 days. We also calculated VE against severe COVID-19, identified through notification to SIVEP-Gripe or with a register of hospitalisation or death in the e-SUS record. Controls were defined as all women in the study population with a negative RT-PCR test result and no positive RT-PCR test in the previous 90 days or in the subsequent 14 days. The test date was defined as either the date of collecting a respiratory specimen or the date of the case registration (when the test date was missing).

### Exposure definition

The exposure studied was vaccination with CoronaVac. This was classified into partially vaccinated (≥ 14 days after the first dose and before receipt of the second dose at the time of RT-PCR testing) and fully vaccinated (≥ 14 days after the second dose at the time of RT-PCR testing). We also calculated effectiveness in the period < 14 days since vaccination as the vaccine is expected to have no or limited effectiveness in the first 13 days since vaccination. This was used as a test as high effectiveness or increased risk during this period might indicate unmeasured bias or confounding. The reference group for vaccination status was the women who did not receive a first vaccine dose before the date of sample collection.

### Covariates

Several risk factors may be associated with both the likelihood of the exposure (i.e. receiving a vaccine) and the likelihood of receiving an RT-PCR SARS-CoV-2 test. These include age, ethnicity, co-morbidities status, geography location, index of deprivation [25], time (reflecting changes in vaccination policy and disease circulation) and presence of a previous COVID-19 notification as this may be related to vaccination the risk of a second COVID-19 infection. We extracted information on these potential confounders from the e-SUS Notifica.

### Statistical analyses

The test-negative design is a type of case-control study in which the study population consist of the population tested, and controls are selected from those who have a negative test [26]. Accordingly, it was analysed using the standard methods for case-control studies [26, 27]. Logistic regression was used to estimate the odds of vaccination with CoronaVac in RT-PCR test confirmed cases compared with those who tested negative and the odds of vaccination in the severe cases compared to those who tested negative. Individuals only contributed their first positive test result from March 15, 2021 (when the vaccination programme was recommended for pregnant women nationally). Week of RT-PCR test was included in the regression models because of the variations over time in both COVID-19 incidence and vaccine delivery in Brazil. We also adjusted for age (< 20, 20–34, ≥ 35), ethnicity (white, mixed brown, black and others), presence of registered co-morbidities, geography (region) and index of deprivation (quintile). Only those with complete information were included in the adjusted analyses. We estimated the VE as one minus the corresponding odds ratio (OR), obtained from a model including the described covariates, expressed as a percentage. Numbers were not sufficient so far to estimate interactions with time since vaccination or variants. We calculated the number of expected deaths in vaccinated women by applying the case fatality rate among the unvaccinated cases to the vaccinated cases, assuming age and presence of co-morbidities were similar.

Data analyses were performed in Stata version 17.0.

This study analysed de-identified data and was approved by the National Ethics committee (CONEP) (CAAE registration no. 50199321.9.0000.0040).

## Results

**Fig. 1:**
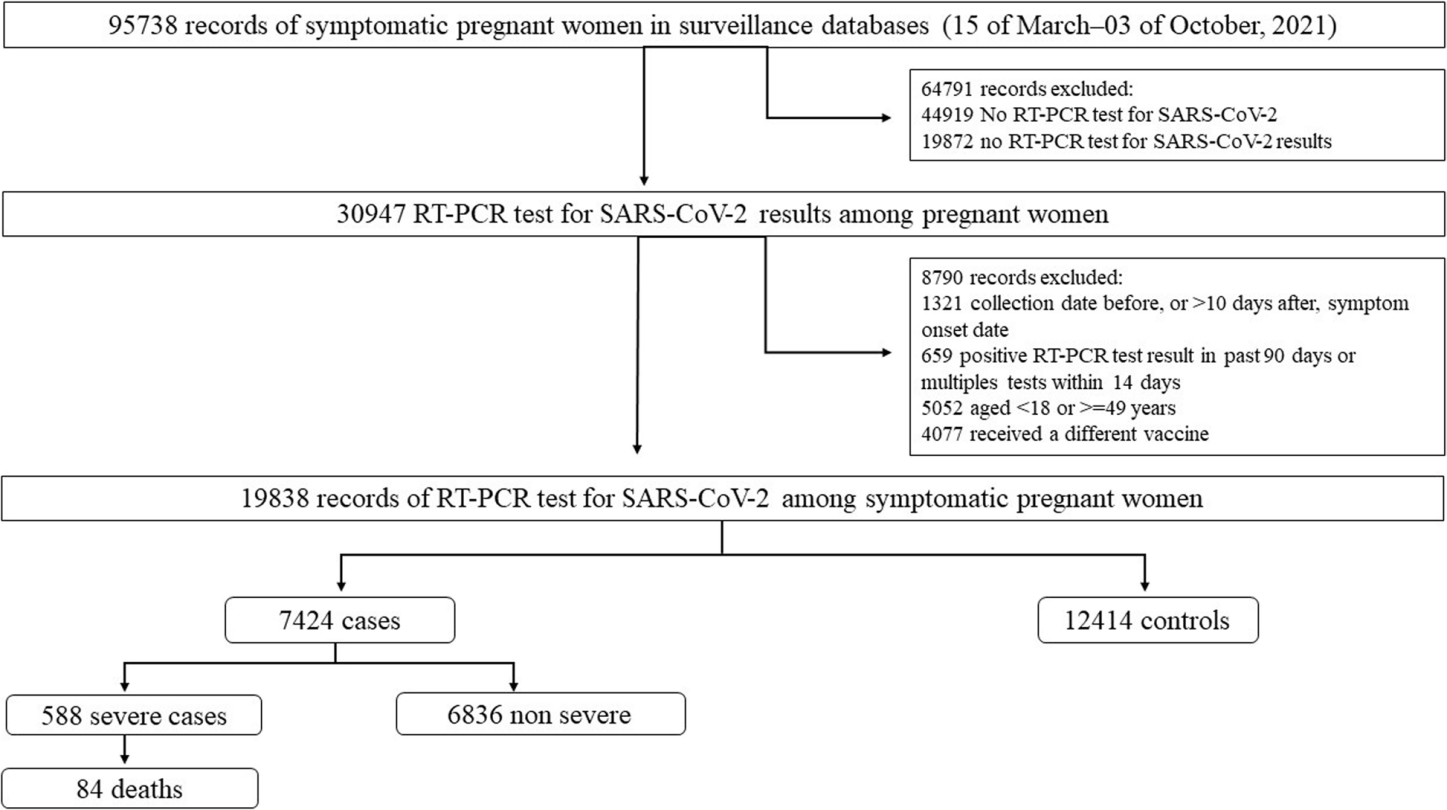
Flowchart of the study population from surveillance system and final sample of cases and controls. RT-PCR, real-time reverse transcription polymerase chain reaction

During the study period, 95,738 symptomatic suspected cases of COVID-19 among pregnant women were registered in the Brazilian surveillance system e-SUS Notify. Of those, 50,819 (53.1%) had an RT-PCR SARS-CoV-2 test, and the results were available for 30,947 (60.9%) samples. After exclusions, 19,838 subjects were included in the analysis: 7424 (37.4%) were test-positive, and 12,414 (62.6%) were test-negative. Of the 7424 with a positive test, 588 (7.9%) were severe, and 84 (1.1%) died (Fig. 1). Table 1 shows the characteristics of the cases and controls. Characteristics more frequent in cases are those that increase the risk of COVID-19: being unvaccinated (92.7%) vs controls (test negative 88.0%), being older and having co-morbidities. Notably, 165 (16.6%) out of all women with a single dose of CoronaVac had not received a second dose after the recommended interval between doses (4 weeks).

**Table 1:** Characteristics of cases and controls in pregnant women aged 18–49 years in Brazil

Characteristics	Test positive	Test negative
**Vaccination status**		
Not vaccinated	6886 (92.75)	10,919 (87.96)
Single dose, within 0-13 days	169 (2.28)	284 (2.29)
Single dose, ≥ 14 days	156 (2.10)	386 (3.11)
Two doses, within 0–13 days	45 (0.61)	192 (1.55)
Two doses, ≥ 14 days	168 (2.26)	633 (5.10)
**Age group**		
< 20	406 (5.47)	940 (7.57)
20–34	5606 (75.51)	9629 (77.57)
35+	1412 (19.02)	1845 (14.86)
Missing	–	–
**Self-reported race**		
White	2787 (43.75)	5226 (47.93)
Mixed Brown	3085 (48.43)	4830 (44.30)
Black	390 (6.12)	689 (6.32)
Others	108 (1.70)	158 (1.45)
Missing	1054	1511
**Reported co-morbidities**		
Yes	554 (7.46)	767 (6.18)
No	6870 (92.54)	11,647 (93.82)
Missing^a^	–	–
**Previous COVID-19-like event notified to surveillance**		
Yes	2447 (32.96)	5145 (41.45)
No	4977 (67.04)	7269 (58.55)
Missing	–	–
**Brazilian Deprivation Index**		
1 (most deprived)	1940 (26.13)	3634 (29.29)
2	1638 (22.07)	2949 (23.77)
3	1502 (20.23)	2269 (18.29)
4	1293 (17.42)	2039 (16.43)
5 (less deprived)	1050 (14.15)	1518 (12.23)
Missing	1	5
**Region of residence**		
North	349 (4.70)	623 (5.02)
Northeast	1663 (22.40)	2244 (18.08)
South	734 (9.89)	2136 (17.21)
Southeast	3981 (53.62)	6444 (51.92)
Midwest	697 (9.39)	965 (7.77)
Missing	–	2

^a^Those who reported only pregnancy as a condition were considered without co-morbidities

The odds of testing positive among those receiving only the first dose with at least 14 days between the first dose and the date of RT-PCR (partially vaccinated) was 0.94 (95% CI 0.76–1.18). Therefore, the VE in this group was low and not statistically significant 5.02 (95% CI −18.22–23.69). For those receiving two doses with at least 14 days between the second dose and the date of RT-PCR (fully vaccinated), the odds of testing positive was 0.59 (95% CI 0.47–0.72). The estimated adjusted VE in the fully vaccinated group against symptomatic COVID-19 was 41.0% (95% CI 27.1 to 52.2) (Table 2).

**Table 2:** Effectiveness of CoronaVac against symptomatic and severe COVID-19, among pregnant women aged 18–49 years in Brazil (comparison of symptomatic and severe cases with test-negative controls)

Sinovac-CoronaVac					
Vaccination status	Unadjusted odds ratio (95% CI)	Unadjusted^**#**^ odds ratio (95% CI)	Adjusted odds ratio (95% CI)	Adjusted* VE% (95% CI)	***p***-value
**Symptomatic COVID-19**					
Unvaccinated	Ref	Ref	Ref	Ref	
One dose < 13 days	0.94 (0.77–1.14)	1.35 (1.10–1.66)	1.35 (1.09–1.68)	–	0.006
Partially vaccinated (one dose ≥ 14 days)	0.64 (0.53–0.77)	1.00 (0.82–1.22)	0.94 (0.76–1.18)	5.02 (–18.22– 23.69)	0.645
Two doses ≥ 14 days	0.42 (0.35–0.50)	0.69 (0.57–0.83)	0.59 (0.47–0.72)	40.97 (27.07– 52.22)	< 0.001
**Severe COVID-19**					
Unvaccinated	Ref	Ref	Ref	Ref	
One dose < 13 days	1.38 (0.87–2.19)	1.64 (1.01–2.65)	1.42 (0.83–2.43)	–	0.192
Partially vaccinated (one dose ≥ 14 days)	0.30 (0.13–0.69)	0.38 (0.16–0.87)	0.32 (0.13–0.80)	67.74 (20.00–87.00)	0.015
Two doses ≥ 14 days	0.15 (0.06–0.37)	0.20 (0.08–0.50)	0.14 (0.05–0.40)	85.39 (59.44– 94.80)	< 0.001

*VE* Vaccine effectiveness

^*^Adjusted for: Age, race, co-morbidities, region of residency, IBP and time

^#^Adjusted for time

The adjusted estimate for severe COVID-19 was an odds ratio of 0.32 (95% CI 0.13–0.80), corresponding to 67.7 (95% CI 20.0–87.0) VE for those partially vaccinated. The adjusted odds ratio for those fully vaccinated was 0.14 (95% CI 0.05–0.40), and vaccine effectiveness was 85.4 (95% CI 59.4–94.8) for fully vaccinated women (Table 2). No deaths occurred among partially or fully vaccinated pregnant women when four would have been expected.

The odds of testing positive among vaccinated women during the 13 days after the first dose was 1.35 (95% CI 1.09–1.68) compared with those unvaccinated. The corresponding estimate for severe COVID-19 was 1.42 (0.83–2.43), indicating an unexpected increase in the risk of COVID-19 and severe COVID-19 among the vaccinated during this initial period.

## Discussion

In this investigation of CoronaVac VE in pregnant women, we found that a single dose of the CoronaVac vaccine offered no protection against symptomatic COVID-19; two doses were 41% effective against symptomatic COVID-19 and 85% effective against severe COVID-19. No deaths occurred among partially or fully vaccinated women when four were expected. About 17% of vaccinated women did not get a second dose as prescribed by the time they were tested.

We found that two doses of CoronaVac administered in pregnant women were overall effective against symptomatic COVID-19 of 41%. This estimate is much lower than the effectiveness of 78 to 96% reported for pregnant women who received the mRNA vaccine BNT162b2 in Israel [9, 10]. CoronaVac VE has varied in different settings. The VE observed in this study is also lower than efficacy estimated in the general population in Chile [5] and Turkey studies [28] but comparable with effectiveness of 53% [29] reported for the general population, 47% [8] from elderly people and 38% among health care workers in Brazil [30]. However, this last group is highly exposed to COVID-19; therefore, vaccine effectiveness may be lower. The difference between our findings and those in the literature might be a result of several factors, including the interval between doses, risk of infection in the community, the predominant variant circulating during the study periods or pregnancy status. Pregnancy promotes resistance to generating proinflammatory antibodies compared to non-pregnant women, suggesting that pregnant women may not respond to some vaccines as effectively [31, 32]. We did not investigate biological mechanisms; further investigation is required to establish whether the lower effectiveness found is due to immunological changes during pregnancy. In contrast with other COVID-19 vaccines, such as the BNT162b2, which confers protection after the first dose [33], CoronaVac was effective against symptomatic COVID-19 only after a complete regimen. This was also found in the older population in Brazil [8]. Since case fatality in pregnancy is higher than in the general population, the same level of protection would prevent more deaths in pregnant women.

This study has strengths and limitations. As a strength, it used rich, routinely collected data from Brazil, recognised as high quality [34]. By using the TND, we have minimised bias related to access to health care, the occurrence of symptoms and health-seeking behaviour. In most populations, strong pressures have influenced who got tested for COVID-19. These biases can mean that those who get tested and test positive for SARS-CoV-2 may not be a random sample of all cases in the population. The assumption that underlies the TND is that people who seek testing and manage to get tested would be influenced by similar pressures regardless of vaccine status and the test outcome [26]; thus, biases will ‘cancel out’, and relatively unbiased estimates of effect can be obtained [26, 27].

On the other hand, our study is subject to potential residual confounding and bias as is the case in most observational studies. The fact that the risk of COVID-19 increased in vaccinated women in the 2 weeks after the first dose is not biologically plausible and may indicate residual bias/confounding, which could lead to an underestimation of VE. A potential explanation for this would be if vaccinated subjects feel safer than unvaccinated subjects, such that unvaccinated subjects are more likely to seek testing for a symptom (not caused by COVID-19) that would not lead a vaccinated subject to test. This would result in a higher proportion of negative tests among the unvaccinated, leading to an apparent estimated increase in risk in the vaccinated, underestimating VE. Other potential explanations are that the process of accessing vaccination itself, e.g. use public transport, increased the risk of infection, or that recently vaccinated women, believing themselves to be reduced protective measures, leading to a peak of infection shortly after vaccination. A limitation intrinsic to the use and availability of secondary data is the limited choice of covariates and the potential for misclassifying vaccine status due to linkage failure.

We did not assess vaccination safety as data necessary for this assessment was not available. However, the adjuvant used in CoronaVac is commonly used in many other vaccines, such as against Hepatitis B and Tetanus, with a well-documented safety profile among pregnant women [35]. Previous evidence of the safety of inactivated vaccines for other pathogens and using this adjuvant is reassuring [35].

We note that an alarming 17% of the study sample with a single dose of CoronaVac did not take the second dose after the recommended maximum interval (4 weeks). This has important repercussions for public health authorities, highlighting the importance of actively searching those delaying the second doses and promoting opportunities to vaccinate these women during regular prenatal care appointments. It is also important to explore the VE for the BNT162b2 vaccine among pregnant women in Brazil. If the results were similar to those observed in Israel, it seems reasonable to offer pregnant women vaccination using mRNA vaccines, followed by inactivated vaccines as the second option. Efficacy must continue to be monitored, in pregnant women as well as other groups, as new variants occur.

## Conclusions

This study involved pregnant women in a setting that combines high disease burden and elevated COVID-19-related maternal related deaths. In this setting, we found that a complete regimen of CoronaVac was 41% effective in preventing symptomatic COVID-19 and 85% effective in preventing severe COVID-19 disease in pregnant women.

## Supplementary Information

**Additional file 1. Table S1.** Vaccination plan in Brazil.

## Data Availability

Importantly, restrictions apply to the availability of these data. Our agreement with MoH for accessing the databases patently denies authorisation of access to a third party. Any request for assessing the databases must be addressed to the Brazilian MoH.

## Abbreviations

CONEP: National Ethics Committee
TND: Test-negative design
SUS: Universal Public Health System (Sistema Único de Saúde)
VE: Vaccine effectiveness
