# Supplementary material for: CoronaVac vaccine is effective in preventing symptomatic and severe COVID-19 in pregnant women in Brazil: a test-negative case-control study
Source: BMC Med. 2022 Apr 5;20:146. doi: 10.1186/s12916-022-02353-w (PMC8979723; doi:10.1186/s12916-022-02353-w)
Supplement: Supplementary file 1 — Additional file 1. Table S1. Vaccination plan in Brazil. [file 12916_2022_2353_MOESM1_ESM.doc]

**Additional file 1**

**Table S1:** Vaccination plan for pregnant and postpartum women in Brazil

| **Date** | **Technical notes issued by the Ministry of Health** | **Recommendations** |
| --- | --- | --- |
| 15/03/2021 | **NOTA TÉCNICA Nº 1/2021-DAPES/SAPS/MS -** Vaccination for pregnant and postpartum women with comorbities | - Vaccination for pregnant and lactating women with co-morbidities  - Vaccine can be offered to pregnant and postpartum women without co-morbidities after evaluating the risks and benefits, especially considering the professional activity performed by the woman. |
| 26/04/2021 | **NOTA TÉCNICA Nº 467/2021-CGPNI/DEIDT/SVS/M**S - Vaccination for pregnant and postpartum women without co-morbidities | Phase I- Pregnant and postpartum women with co-morbidities, regardless of age  Phase II- Pregnant and postpartum women, regardless of co-morbidities |
| 14/05/2021 | **NOTA TÉCNICA nº 627/2021-CGPNI/DEIDT/SVS/MS** - Temporary suspension of vaccination | - Temporary suspension of vaccination with the vaccine AstraZeneca/Oxford/Fiocruz in pregnant and postpartum women |
| 19/05/2021 | **NOTA TÉCNICA Nº 651/2021 - CGPNI/DEIDT/SVS/MS - C**ontinued vaccination in pregnant and postpartum women with co-morbidities | - Vaccination of pregnant and postpartum women with co-morbidities after benefit risk evaluation and medical prescription (Vaccines without viral vector -SINOVAC/Butantan or Pfizer-BioNTech BNT162b2)  - Pregnant and postpartum women (including those without additional risk factors) who have already received the first dose of the AstraZeneca/Oxford/Fiocruz vaccine must wait for the end of the gestation and postpartum period (up to 45 days after delivery) for the administration of the second dose of the vaccine  - Pregnant and postpartum women (including those without additional risk factors) who have already received the first dose of another COVID-19 vaccine that does not contain a viral vector (Sinovac/Butantan or Pfizer-BioNTech BNT162b2) should complete the regimen with the same vaccine at the usual intervals  - Pregnant and postpartum women of other priority groups (health workers or other essential services workers, for example) may be vaccinated after an individual risk and benefit evaluation |
| 06/07/2021 | **NOTA TÉCNICA Nº 2/2021 - SECOVID/GAB/SECOVID/MS -** Continued vaccination in pregnant and postpartum women without co-morbidities | - Vaccination of pregnant and postpartum women aged 18 years and over, regardless of risk factors  - Pregnant of any gestational age  - Needs for Medical evaluation and Prescription |
| 23/07/2021 | **NOTA TÉCNICA Nº 6/2021-SECOVID/GAB/SECOVID/MS** - Interchangeability between vaccines for pregnant and postpartum women who took the oxford astrazeneca vaccine in the first dose | - Vaccination of pregnant and postpartum women aged 18 years and over, regardless of risk factors  - Pregnant of any gestational age  - Need for Medical evaluation and Prescription  - To pregnant and postpartum women who received the first dose of the AstraZeneca/Fiocruz vaccine, at time of the second dose, preferably, the Pfizer-BioNTech BNT162b2 /Wyeth vaccine should be offered. If this immunising agent is not available locally, Sinovac/Butantan vaccine may be used |
